# A Manual of Immunity

**Published:** 1914-03

**Authors:** 


**A Manual of Immunity.** By ELIZABETH T. FRASER, M.D. Pp. x, 199. Glasgow: James Maclehose & Sons. 1912. Price 5s. net.

This little book of 200 pages will be found an extremely useful résumé of those aspects of the theory of immunity which are of greatest practical importance to the diagnostician and practitioner. After a brief historical survey, the author plunges *in medias res*, and in terse and lucid language deals in succession with the body fluids, the cell, and the micro-organism as factors in immunity, and then with immunity-reactions employed for therapeutic ends and for diagnostic purposes, ending with a chapter on anaphylaxis. The kindred topics of salvarsan and neo-salvarsan are briefly treated in an appendix. To what extent the writer draws on personal experience in her descriptions of diagnostic processes and results is not evident, and is just a point on which one would like more information. For example, the modifications of the Wassermann test are dismissed with the remark that none are thoroughly trustworthy. The reviewer has made personal trial of the Fleming modification in many hundreds of selected cases, with the clinical appearances and histories available for comparison, and an analysis of the results convinces him that, carried out conscientiously, and as Fleming now uses it, the results are of great practical utility, whilst the comparative simplicity of the method enables one to employ it in scores of cases which otherwise would be neglected from this point of view.

